# Editorial: Neural substrates of acupuncture: From peripheral to central nervous system mechanisms, volume II

**DOI:** 10.3389/fnins.2022.1119829

**Published:** 2023-01-19

**Authors:** Younbyoung Chae, Florian Beissner, Hee-Young Kim, Richard E. Harris, Vitaly Napadow

**Affiliations:** ^1^Acupuncture and Meridian Science Research Center, Kyung Hee University, Seoul, Republic of Korea; ^2^Insula Institute for Integrative Therapy Research, Hanover, Germany; ^3^Department of Physiology, Yonsei University College of Medicine, Seoul, Republic of Korea; ^4^Department of Anesthesiology, Chronic Pain and Fatigue Research Center, University of Michigan, Ann Arbor, MI, United States; ^5^Athinoula A. Martinos Center for Biomedical Imaging, Massachusetts General Hospital, Charlestown, MA, United States; ^6^Department of Physical Medicine and Rehabilitation, Spaulding Rehabilitation Hospital and Harvard Medical School, Charlestown, MA, United States

**Keywords:** acupuncture, biomarker, neuroscience, somatosensation, acupoints

During the past several decades, acupuncture therapy has been extensively studied and shown to be clinically effective for a variety of illnesses, particularly pain disorders (Lee et al., 2020). The efficacy and mechanism of action of acupuncture are two key issues. In fact, a large number of clinical trials have been conducted to investigate the efficacy of acupuncture treatment (Vickers et al., 2018). To clarify the underlying mechanisms of acupuncture, numerous mechanistic investigations have been performed using both human and non-human (i.e., rodent) pre-clinical models (Han, 2004; Moffet, 2006; Napadow et al., 2008; Chae et al., 2013). While our understanding of acupuncture mechanisms has certainly improved, multiple questions, such as problem of acupoint specificity, has not yet been fully understood (Xing et al., 2013; Langevin and Wayne, 2018).

The World Health Organization has proposed a standardized nomenclature and locations for acupoints, but variation is still seen among practitioners (Molsberger et al., 2012; Godson and Wardle, 2019). Although numerous studies have investigated the anatomical features and electrical properties of acupoints (Langevin et al., 2002; Ahn et al., 2008, 2010; Soh, 2009; Lee et al., 2019), they remain to be fully elucidated (Colbert et al., 2011), and more research is needed. This research will include novel explorations as well as the synthesis of existing information on acupoints and biomarkers in databases and the published literature.

The current issue includes six articles on the state of research on neural substrates of acupuncture. Three original studies investigated the neural circuitry underlying electro-acupuncture for cardiovascular diseases, functional connectivity alterations associated with moxibustion for primary dysmenorrhea, and neurobiological markers for electroacupuncture in an animal irritable bowel syndrome model. In addition, three review articles are also included and explore the neural pathways and biophysical features of acupoints, along with neurobiological markers of sham acupuncture—a form of control intervention commonly used in clinical trials.

By balancing the somato-sympathetic and the somato-parasympathetic reflexes, electro-acupuncture at ST25 reduced visceral hypersensitivity in an animal model of irritable bowel syndrome (Zhang et al.). Electroacupuncture applied to PC5-PC6 acupoints (innervated by median afferents) was found to control a cardiopulmonary reflex through the hypothalamic paraventricular nucleus (Tjen-A-Looi et al.). In another human brain imaging study, moxibustion at CV4, CV8, and SP6 modulated pain in dysmenorrhea by altering the connectivity between the inferior frontal gyrus and default mode network involved in the pain reappraisal and processing (Yang et al.). Based on these results, we speculate that specific acupoints can modulate neural pathways to exert various effects on visceral function.

Regarding acupoint characteristics, Cui et al., investigated the functions of cutaneous C nociceptors to understand acupoint sensitization and plasticity. Additionally, Lee et al., posited that myofascial trigger points are closely related to so-called “Ashi” points, and proposed a relationship among classical acupuncture points, extra-acupuncture points, and Ashi points. Furthermore, based on the outcomes of 51 sham acupuncture experiments, Kim et al., reported that sham acupuncture, as commonly used in acupuncture RCT's, had similar effects on biomarkers to acupuncture.

We hope that readers will enjoy this issue of *Frontiers in Neuroscience*, which expands on the neurological mechanisms of acupuncture and fundamental issues regarding acupuncture points and placebo controls for clinical trials. We believe that these findings may help establish the link between clinical acupuncture therapy and basic and translational research.

## Author contributions

All authors listed have made a substantial, direct, and intellectual contribution to the work and approved it for publication.
